# Azimuthally-variant perfect vector beams for the control of arbitrary phase and polarization ring patterns

**DOI:** 10.1038/s41377-025-01859-1

**Published:** 2025-05-06

**Authors:** Andrea Vogliardi, Gianluca Ruffato, Daniele Bonaldo, Simone Dal Zilio, Filippo Romanato

**Affiliations:** 1 Department of Physics and Astronomy ‘G. Galilei’, University of Padova, Padova, Italy; 2 Padua Quantum Technology Research Center ’QTech’, University of Padova, Padova, Italy; 3 Department of Information Engineering, University of Padova, Padova, Italy; 4 Istituto Officina dei Materiali, CNR, Trieste, Italy

**Keywords:** Metamaterials, Nanophotonics and plasmonics

## Abstract

Perfect vortices, recognized for their distinct ring profile that remains independent of the topological charge, present significant challenges in generation due to the precise control needed over both phase and polarization. In this work, we introduce and validate a new approach for generating these beams, allowing the selection of different azimuthally-variant phase gradients and vector states, thereby enabling full control over the phase and polarization patterns of perfect vortices. Using dual-functional silicon metaoptics, we achieve the compact generation of a novel class of perfect vortices, termed azimuthally-variant perfect vector beams. The optical characterization of the generated beams, performed through a filtering method, confirms their intrinsic azimuthally-variant vectorial nature. These beams exhibit unique properties that promise valuable applications in optical tweezing, the manipulation of low-refractive-index particles, the trapping of cold atoms, and high-capacity communications.

## Abstract (web summary)

Dual‐functional metaoptics generating novel perfect vector beams with tailored phase and polarization via dynamic azimuthally‐variant gradients.

## Graphical abstract

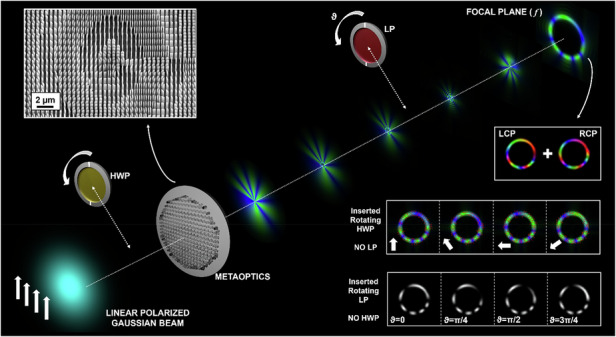

## Introduction

Since Coullet et al. introduced the concept of optical vortices in 1989^1^, the domain of structured light^2^ has enhanced the understanding of various optical phenomena and prompted significant advancements in numerous fields, in addition to the increased versatility and integration in light manipulation technologies^3^.

Actually, the capability to engineer optical beams has progressed along two complementary directions. On the one side, the development of sophisticated tools and theoretical models aimed at precisely controlling the spatial configuration of various degrees of freedom within the beam, including amplitude, phase, polarization, and orbital angular momentum (OAM)^4^. In parallel, research has focused on the advancement of cutting-edge optical elements, characterized by progressively higher levels of integration and increased functional density, designed to leverage the properties of complex light and pursue integration into existing optical and photonic platforms^5^.

Over the past few decades, beams carrying orbital angular momentum have emerged as a paradigmatic example of this synergy, exemplifying the profound interplay between our advancing understanding of light fundamental properties and the concurrent development of innovative optical technologies.

Indeed, despite marking disruptive advancements in multiple disciplines, from life science to information and communication technology^6–11^, the intensity distributions of standard OAM beams, such as Bessel^12^, Kummer^13^, and Laguerre-Gaussian^14^ were intrinsically related to their twisting phase structure, limiting the full exploitation in trapping and spatial division multiplexing^15,16^.

To address this challenge, Ostrovsky et al.^17^ first introduced the concept of perfect vortices, showing the possibility of encoding any azimuthal phase onto a fixed intensity ring having the desired radius and width, via the Fourier transform of axicon and spiral phase plate contributions^18^. Concurrently, the control over polarization ignited further the field, showing the potentiality of its combination with orbital angular momentum^19,20^ in imaging^21^, lithography,^22^, rotation-invariant systems^23^, and high-dimensional quantum communications^24,25^.

Although the concept of vectoriality is theoretically applicable to any structured beam^26^, achieving a non-separable combination of spin and orbital angular momenta presented significant challenges when using conventional optics. Such combinations typically required complex setups involving multiple optical elements or interferometric architectures^27^, limiting their practical implementation and widespread adoption. Introducing unprecedented manipulation of light at the subwavelength scale, metasurfaces enabled the combined control of phase and polarization into the same platform, providing a key enabling technology for the compact generation of vector beams in many areas such as optical communications, quantum optics, and biomedical imaging. Moreover, this accelerated further the progress in OAM-beam shaping and applications. While the possibility to generate multiple rings with different vorticity^28^ extended the applications to information technology^29–33^ and trapping of low refractive index particles^34,35^, a first attempt to go beyond the standard azimuthal phase was done by uploading multiple topological charges via grafted phase design^36–38^. Nevertheless, the approach does not allow for complete control point-by-point of the phase and polarization along the ring.

In this work, we demonstrate for the first time the generation of a new class of beams offering complete control over both phase and polarization through arbitrary azimuthal functions. These beams called azimuthally-variant perfect vector beams (AV-PVBs), enable compression or stretching of phase and polarization based on predefined functions embedded during design. Dual-functional metasurfaces become essential to provide the polarization-sensitive phase shaping required for the spin-decoupled manipulation of an input linearly polarized beam, encoding distinct phase patterns onto circularly polarized components^39^. This approach generates non-separable combinations of spin and orbital angular momentum using a single optical element, while simultaneously imparting the polarization-insensitive phase pattern for the desired vortex beam following any azimuthally variant phase distribution. The designed optical elements are realized using optimized nanofabrication techniques on a silicon substrate, enabling precise replication of the meta-atoms at the nanoscale and accurate generation of the phase map profile. Optical characterizations confirm the capability to generate rings with continuously-variant arbitrary phase profiles and polarization patterns, suggesting novel and smart optical elements for beam shaping and light control with unprecedented levels of compactness and integrability.

## Results

### Theory

In recent years, the focus of Orbital Angular Momentum (OAM) beam generation has shifted towards perfect vortices, which offers the advantage of a customizable ring structure that remains independent of the OAM they carry. These perfect vortices can be generated by illuminating a spiral axicon with a Gaussian beam, effectively representing the Fourier transform of a high-order Bessel-Gaussian beam, as detailed in studies like^17,18^.

Metasurfaces consolidate the functions of several bulky optical elements into a single, flat optic, offering an advanced design strategy by discretizing and layering distinct phase profiles onto one metasurface. The design of a metalens that enables the generation of perfect vortices with opposite orbital angular momentum at a designated focal plane is determined by a single phase map, which can be represented as the sum of multiple contributions, as follows^40^:

1 $${\Phi }^{\pm }={\Phi }_{\pm \ell }+{\Phi }_{axicon}+{\Phi }_{lens}$$

Here, ${\Phi }_{\pm \ell }=\pm \ell \varphi$ represents the Orbital Angular Momentum (OAM) which, with a staircase phase profile, transfers $\pm \ell$ units of ℏ per photon^41^. ${\Phi }_{axicon}=-\alpha r$ describes the phase profile associated with the axicon, while ${\Phi }_{lens}=-k\left(\sqrt{({r}^{2}+{f}^{2})}-f\right)$ providing the geometrically aberration-free focusing phase profile, where *f* is the focal length and $k=2\pi /\lambda$ is the wavenumber^42,43^.

In the focal plane of the metalens, an OAM beam is produced, characterized by a ring-shaped intensity profile whose radius and width depend on the waist of the input beam (*w*_0_), the wavenumber (*k*), and the lens and axicon parameters (*α* and *f*)^40^.

To enable the generation of azimuthally-variant phase delays, allowing precise control over phase and polarization patterns along the ring, the momentum-dependent term ${\Phi }_{\pm \ell }$ is extended to a new phase term ${\Phi }_{m,{\ell }_{0},{\ell }_{1}}$, which governs the azimuthally-variant phase gradient:

2 $${\Phi }_{m,{\ell }_{0},{\ell }_{1}}^{\pm }=\mathop{\sum }\limits_{j=1}^{m}{(-1)}^{{H}_{C}^{j}}[\pm ({\ell }_{0}+{H}_{G}^{j}{\ell }_{1})\pm {(-1)}^{{H}_{G}^{j}}({\ell }_{1}-{\ell }_{0})\beta (m\cdot \phi )]\phi$$

where $j\frac{2\pi }{m} \,<\, \phi \,<\, (j-1)\frac{2\pi }{m}\,\forall j=1,{..}.,m$, $\beta (\cdot )$ is the function that imparts the azimuthally variant phase gradient, *m* represents how many parts of the ring we decide to encode this variation, *ℓ*_0_ is the background topological charge, *ℓ*_*1*_ is a parameter that is responsible for both the amplitude of the phase gradient along one of the *m* sectors and of the overall topological charge of the beam. In this equation, we have to satisfy the condition *ℓ*_0_ < *ℓ*_1_. The two Heaviside functions *H*_*G*_ and *H*_*C*_ enable, respectively, the inversion of the azimuthal gradient sign and the inversion of the topological charge sign in each *j-th* sector.

The generated beams have an overall topological charge arising from the azimuthally-variant phase gradient that can be exploited also for the design and generation of vector beams distribution as a result of non-separable combinations of polarization states and spatial modes (refs. ^19,44^) (see Supplementary Material).

Dual-functional metalenses (DFMLs), capable of independently manipulating orthogonal circularly polarized beams, are well-suited for generating vector beams (see Supplementary Material) when illuminated by a linearly polarized beam. The relative Jones vector is given by:

3 $$J|\theta \rangle =u(r)\frac{{e}^{i\theta }{e}^{i{\Phi }^{-}}|L\rangle +{e}^{-i\theta }{e}^{i{\Phi }^{+}}|R\rangle }{\sqrt{2}}$$

where *u*(*r*) is the amplitude term unrelated to the topological charge and polarization handedness, |*L*〉 and |*R*〉 represent the Jones vector of left-handed and right-handed circularly polarized components, *θ* is the angle of the illuminating linearly polarized beam, and Φ^±^ are the phase delays imparted to the two orthogonal circular components. By substituting the phase pattern of Eqs. (1, 2) in the DFMLs paradigm (Eq. 3), it turns out that the output beam is endowed with a spatially variable polarization pattern $\theta ^{\prime} =\theta -|{\Phi }_{m,{\ell }_{0},{\ell }_{1}}^{\pm }|$ over the intensity distribution *u*(*r*) which is purely modulated by the

impinging beam, Φ_*axicon*_, and Φ_*lens*_. At the focal length $u(r)={e}^{-{(\frac{r-{\rho }_{0}}{d{\rho }_{0}})}^{2}}$ where ${\rho }_{0}=\frac{\alpha f}{k}$ and $d{\rho }_{0}=\frac{2f}{k{w}_{0}}$^40^. If the phase pattern ${\Phi }_{m,{\ell }_{0},{\ell }_{1}}^{\pm }$ were flipped in the design, the resulting polarization pattern would be symmetric $\theta ^{\prime} =\theta +|{\Phi }_{m,{\ell }_{0},{\ell }_{1}}^{\pm }|$ but the intensity distribution would remain unchanged^28^. By expanding $|{\Phi }_{m,{\ell }_{0},{\ell }_{1}}^{\pm }|$, the distribution of the polarization pattern along the intensity can be split into two distinct components: one that remains constant with respect to the azimuthal angle (${\theta ^{\prime} }_{0}$) and another that varies azimuthally (${\theta ^{\prime} }_{\phi }$):

4 $$\begin{array}{c}\theta ^{\prime} ={\theta ^{\prime} }_{0}+{\theta ^{\prime} }_{\phi }\,\\ \,=\left(\theta -\mathop{\sum }\limits_{j=1}^{m}{(-1)}^{{H}_{C}^{j}}[+({\ell }_{0}+{H}_{G}^{j}{\ell }_{1})]\right)+\left(\mathop{\sum }\limits_{j=1}^{m}{(-1)}^{{H}_{C}^{j}}[+{(-1)}^{{H}_{G}^{j}}({\ell }_{1}-{\ell }_{0})\beta (m\cdot \phi )]\right)\end{array}$$

**Fig. 1:**
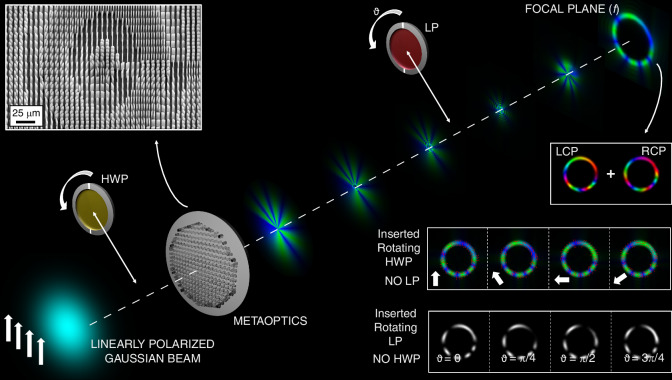
Schematic representation of the AV-PVB generation. When the designed metasurface is illuminated by a linearly polarized Gaussian beam, an AV-PVB is formed at the focal plane, with its phase and polarization characteristics determined by the metasurface design. The perfect vortex is generated by leveraging the bifunctionality of the metaoptics, which imparts distinct phase patterns based on the spin of the circularly polarized light in input. By adjusting the polarization angle of the incoming linearly polarized Gaussian beam using a half-wave plate, the polarization pattern at the focal plane can be modified, as shown by the variations in the blue and green patterns (representing the *x*- and *y*-components of the electric field, respectively). Introducing a rotating linear polarizer after the metasurface (whose principal axis is directed along the direction of the white arrows in the second row) enables the creation of a multi-spot configuration, with spot sizes and rotation velocities or directions that vary according to the polarization pattern

In general, when illuminated with linearly polarized light, it is possible to generate a non-separable combination of polarization and spatial modes, leading to a non-uniform polarization pattern (see Fig. 1).

An extension of the paradigm can be the generation of multiple non-overlapping azimuthally-variant perfect vector beams with equalized intensities but with different characteristics, *i*.*e*., number of rings, polarization states, and topological charges, by introducing the multiple-axicon equation for the phase patterns Φ^±^^28^:

5 $${\Phi }^{\pm }={\text{arg}}\left(\mathop{\sum }\limits_{nr=1}^{N}{A}_{nr}\cdot {e}^{i{\Phi }_{nr}^{\pm }}\right)$$

where *nr* = 1,…, *N* (being *N* is the number of rings), *A*_*nr*_ are the different weights for each perfect vortex, and Φ^±^_*nr*_ is the azimuthally variant phase for each ring. Thus, by imposing the same focal length but different spatial frequencies for the *N* axicon terms *α*_*nr*_, the distance between the intensity rings can be tuned, allowing the generation of multiple concentric rings, each with their proper polarization and topological variant distribution. To determine the orbital angular momentum of our vortex beams, we calculated the topological charge (*Q*) by integrating the beam’s phase along a closed path around the central singularity^41^:6where ${\Phi }_{m,{\ell }_{0},{\ell }_{1}}$ is the only term contributing to the topological charge, as the axicon and lens terms have circular symmetry and do not affect the result. In the simplest scenario of a simple perfect vortex, where *m* = 1, *H*_*C*_^1^ = *H*_*G*_^1^ = 0, and *β*(*m*·*φ*) = *C* (a constant), the topological charge reduces to *Q* = *C*(*ℓ*_1_ − *ℓ*_0_) − *ℓ*_0_, and corresponds to the number of 0-to-2*π* phase steps, while the helicity is determined by the direction of the phase gradient. When *β*(*m* · *φ*) is a function of *φ* and *m* > 1, the solution becomes not trivial and leads to interesting cases such as generating vector beams from two beams in orthogonal polarization states having null topological charge as reported below and more detailed in Supplementary Material.

### Metaoptics encoding linear phase gradients

In order to validate our design approach, we initially designed five distinct optical elements with varying configurations, all encoding linear phase gradients based on the simplest phase gradient function, that is $\beta (\cdot )=\frac{\varphi }{2\pi }$.

**Fig. 2:**
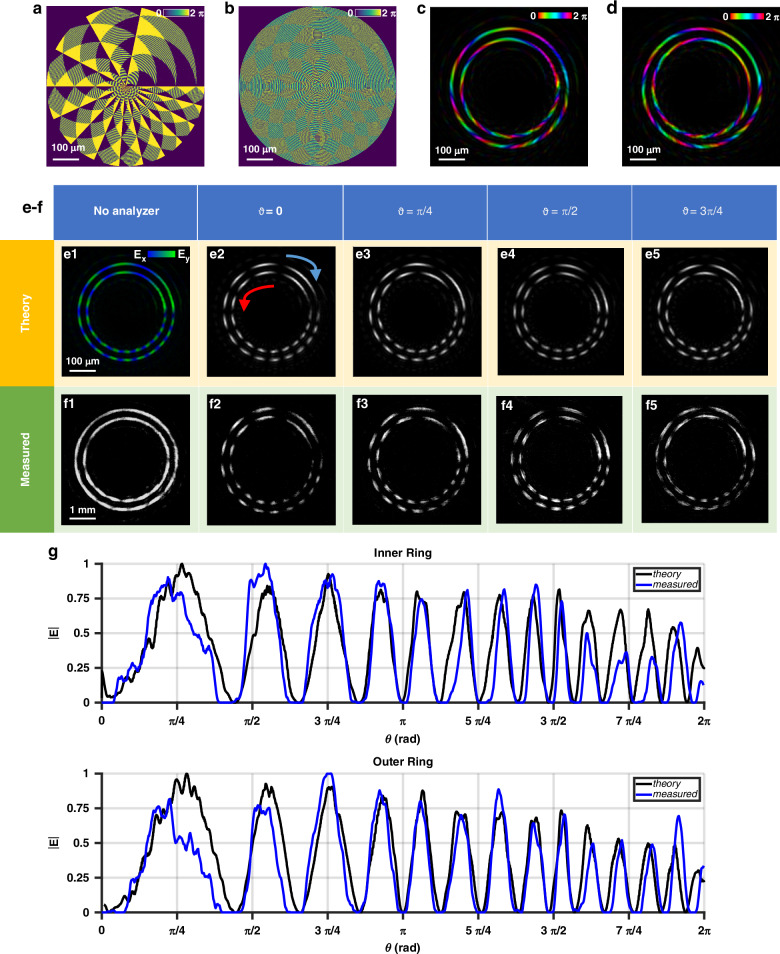
Metaoptics generating two rings having a single gradient sector but opposite vortex states. Encoded dynamic (**a**) and geometric (**b**) phases of the metaoptics. Simulation of the optical response of the metaoptics under the illumination of a Gaussian beam left-handed (**c**) and right-handed (**d**) circularly polarized. Simulated (**e**) and measured (**f**) intensity of the vector beam before (**e1**, **f1**) and after linear polarization filtering (**e2**–**e5**, **f2**–**f5**). The azimuthal phase gradient generates an azimuthal variation of the electric field’s amplitude components (*E*_*x*_ and *E*_*y*_) along the ring (**e1**). Inserting a rotating linear polarizer at different angles, the measured spot size variation (**f2**–**f5**) is compared to the simulation (**e2**–**e5**). The vortex (antivortex) state of the inner (outer) ring generates an anticlockwise (clockwise) rotation. Comparison between theory and simulations of the azimuthal intensity profile after filtering for both rings (**g**) when *ϑ* = *π/*2

The first optical element in this set generates two rings (Fig. 2), both exhibiting the same phase gradient over a single sector, transitioning from *ℓ*_0_ = 0 to *ℓ*_1_ = 7. The dynamic and geometric phases were designed to create opposite vortex states, which display inverse phase gradients and phases when illuminated by Gaussian beams with left- and right-handed circular polarizations. The topological charges of circular components are ±7, which are appreciable from the seven phase jumps in Fig. 2c, d, resulting in a vector beam of order 7. The vectorial nature of the beams is revealed through a rotating linear polarizer, producing azimuthally distributed spots that rotate in opposite directions depending on the polarization handedness. The |2*ℓ*_1_| spots vary in size, proportional to the phase gradient.

Three additional optical elements were designed and fabricated to explore other configurations (Supplementary Material), changing the number of sectors, topological charges, and vectorial states once at a time. In particular, we elucidated how to generate vectorial states from a beam that has no overall topological charge by tuning the design parameters. Moreover, we showed how to produce hybrid-order vector beams using the same paradigm (Supplementary Material).

**Fig. 3:**
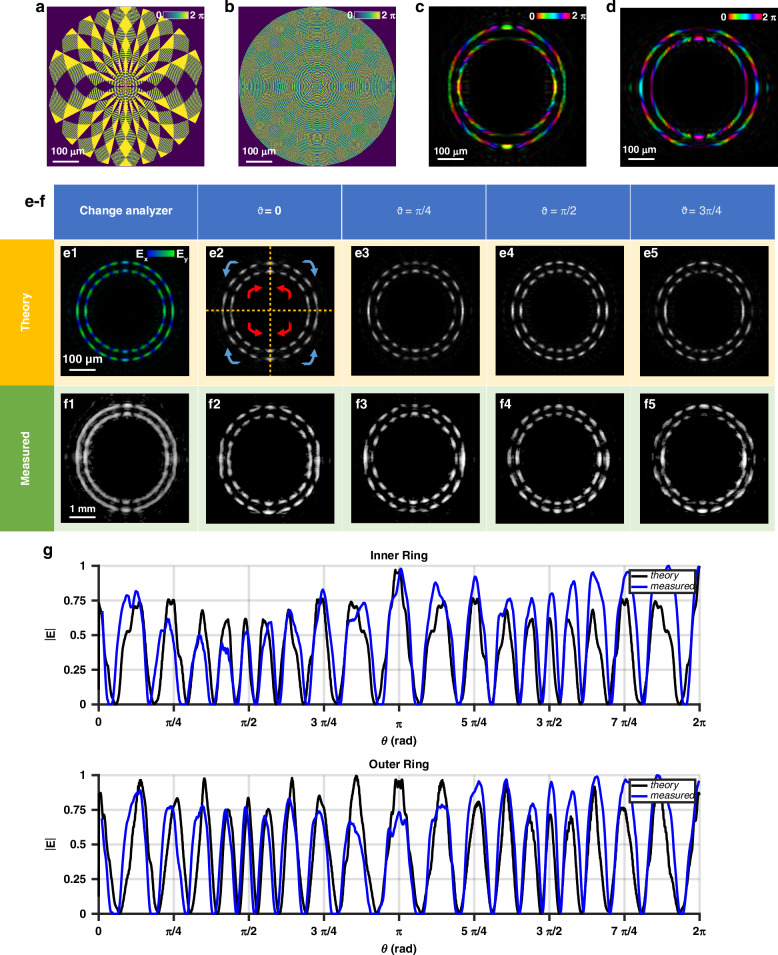
Metaoptics generating two rings divided in four sectors and having null topological charges but arising azimuthally variant polarization pattern. Encoded dynamic (**a**) and geometric (**b**) phases of metaoptics generating two rings in opposite vector states having four gradient sectors with alternating gradient inversion. Simulated optical response of the metaoptics under the illumination of a Gaussian beam left-handed (**c**) and right-handed (**d**) circularly polarized. Simulated (**e**) and measured (**f**) intensity of the vector beam before (**e1**, **f1**) and after linear polarization filtering (**e2**-**e5**, **f2**–**f5**). The azimuthal phase gradient generates an azimuthal variation of the electric field’s amplitude components (*E*_*x*_ and *E*_*y*_) along the ring (**e1**). Inserting a rotating linear polarizer at different angles shows the measured variation of the vector beam spot sizes (**f2**–**f5**) after the filtering to compare with the simulation (**e2**–**e5**). In each of the four sectors, the inner and the outer rings are in opposite vector states, leading to opposite spot rotation. Comparison between theory and simulations of the azimuthal intensity profile after filtering for both rings (**g**) when *ϑ* = *π/*2

In the fifth optics, the vortex and antivortex states were alternately encoded in the inner and outer rings, with gradient inversions applied across four sectors of the double rings, and topological charges set to *ℓ*_0_ = 5 and *ℓ*_1_ = 10 in both rings (Fig. 3). The observed phase gradient and sign inversions under circularly polarized illumination, together with the polarization pattern generated in the vertically polarized light simulation, show that the circular components do not carry an overall topological charge, but the vector beam of order *ℓ*_1_ is correctly generated. The experimental results closely match the theoretical predictions, as shown by the azimuthal intensity profile after filtering. Experimental videos also demonstrate the evolution of the vector beam as the analyzer rotates.

Lastly, the behavior of a metaoptic designed to generate three concentric rings (*N* = 3), each featuring distinct azimuthally-varying linear phase gradients divided into different sectors with unique intrinsic vectorial states, is discussed in Supplementary Material. The structured beam was also generated under horizontally polarized light, confirming the inversion of the vectorial state in each sector.

### Non-linear phase gradient functions

This section investigates the topological vector beam distribution generated by a non-linear phase gradient function, presenting two additional metaoptics. For simplicity, the design consists of a single ring and a single sector without phase gradient inversion and topological charge inversion.

**Fig. 4:**
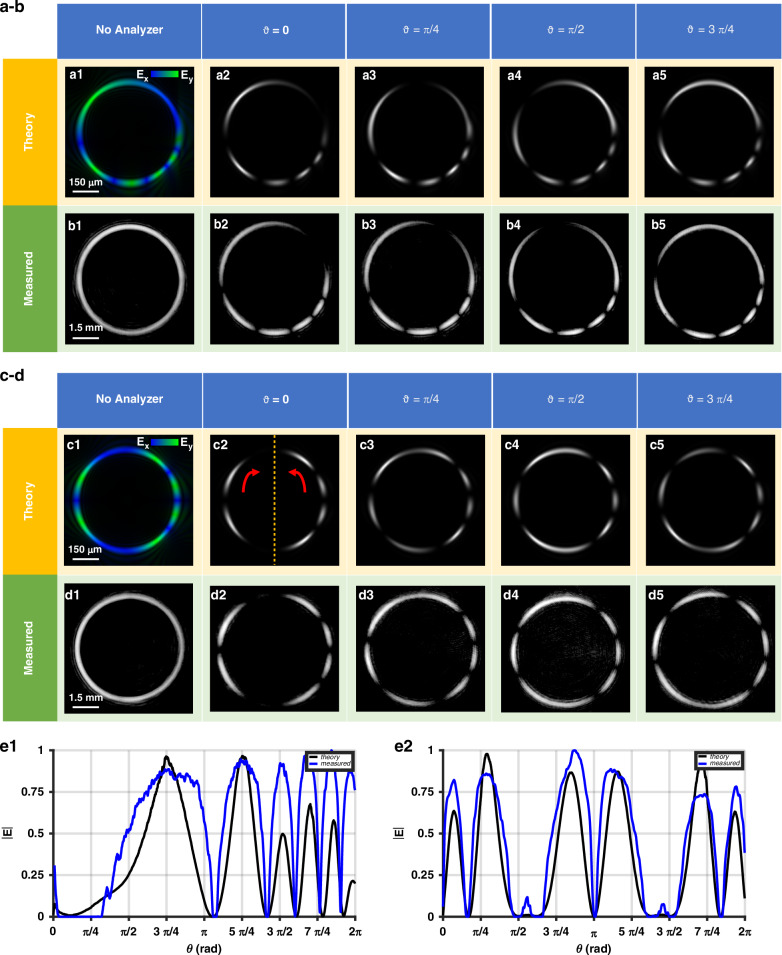
Metaoptics generating AV-PVBs with exponential and sinusoidal phase gradients. Simulated and measured optical response of metaoptics imposing *ℓ*_0_ = 0, *ℓ*_1_ = 3 and the phase gradient function ${\mathbf{\beta}} (\cdot ){\mathbf{=}}\frac{{\mathbf{exp}} ({\mathbf{A}}\cdot \frac{{\mathbf{\phi}} }{{\mathbf{2}}{\mathbf{\pi}} })}{{\mathbf{exp}} ({\mathbf{A}})}$ with *A* = 2.5. Simulated (**a1**) and experimental (**b1**) measure of the generated beam under vertically polarized light. *|*2*ℓ*_1_| variations between *E*_*x*_ and *E*_*y*_ confirm the vectorial nature of the beam. The variation of polarization is weak in the first area of the ring but increases a lot following exponential behavior. Simulated (**a2-5**) and experimental (**b2-5**) results of beam characterization after filtering. The spots rotate in the same direction as the analyzer, as the ring is in the vortex state. Simulated (**c**) and measured (**d**) optical response of metaoptics imposing *ℓ*_0_ = 1, *ℓ*_1_ = 3, and the phase gradient function $\beta (\cdot )=\frac{sin(\varphi )}{\pi \varphi }$. $|2{\ell }_{1}|$ variations between *E*_*x*_ and *E*_*y*_ confirm the vectorial nature of the beam. The variation of polarization is asymmetrical due to the *π* periodicity of the sin(·) function and the contribution of the background charge *ℓ*_0_. Moreover, because of the periodicity mentioned above, the ring is intrinsically divided into two sub-sectors in opposite states as the gradient of the function changes sign every *π*. Simulated (**c2–5**) and experimental (**d2–5**) results of beam characterization after filtering. The spots rotate in the same direction as the analyzer, from −*π/*2 to *π/*2 as the gradient is positive; otherwise, they rotate in the opposite direction. The experimental azimuthal profile fits well with the theoretical one. Comparison between theory and simulations of the azimuthal intensity profile after filtering for both samples (**e**) when *ϑ* = 0

The first metasurface employs an exponential azimuthal phase gradient, $\beta (\cdot )=\frac{\exp (A\cdot \frac{\varphi }{2\pi })}{\exp (A)}$ where *A* is a constant that controls the variation of the exponential gradient and $\frac{1}{\exp (A)}$ is a normalization factor. Selecting *ℓ*_0_ = 0 and *ℓ*_1_ = 3, the two generated orthogonal circularly polarized components have topological charge equal to ±*ℓ*_1_, resulting in an exponential distribution of third-order vector beams, as illustrated by the azimuthal electric field profile (Fig. 4). As expected, after filtering, the |2*ℓ*_1_| spots rotate in the same direction as the analyzer and increase exponentially simultaneously with the variation in the azimuthal coordinate.

A sinusoidal gradient function, $\beta (\cdot )=\frac{\sin \varphi }{\pi \varphi }$, with *ℓ*_0_ = 1 and *ℓ*_1_ = 3, was implemented in a second metaoptics (Fig. 4c, d) that results in an oscillatory asymmetric vector beam. Being *sin*(·) the gradient function, we had an intrinsic oscillation in terms of *ℓ* local contribution leading to a consecutive inversion of the gradient and sign arising a total topological charge of ±2 for the circular components but a vector beam order of *ℓ*_1_ = 3. Thus, periodic functions allow the introduction of different gradient sub-behaviors, complicating the phase and polarization pattern even more.

## Discussion

The reported findings showcase the transformative potential of metasurfaces for generating complex structured light fields, specifically a novel class termed azimuthally-variant perfect vector beams (AV-PVBs). Through meticulous design and nanofabrication, arbitrary and analytical azimuthal functions can be encoded for the first time into compact metaoptics, enabling unprecedented control over the phase and polarization patterns of perfect vortex beams and overcoming the limitations of prior designs based on the combination of discrete values or grafted combinations of topological charges.

A key advantage stems from the dual-functional nature of these metaoptics, enabling parallel operations onto polarization and phase in combination with independent manipulation of orthogonal and circularly polarized components. This capability enables the creation of complex vector beams with customizable polarization distributions along the ring structures, ranging from purely scalar to purely vector, as well as any hybrid combination by adjusting the degree of non-separability (or concurrence that we showed can span the full range^45^) (See Supplementary Material).

A significant outcome is the novel utilization of the input polarization as a dynamic control mechanism for the output beam. In particular, two distinct moving configurations exploit polarization rotation. In one setup, a rotating analyzer placed after the metaoptics selects different polarization states along the ring pattern, resulting in a series of bright spots with varying sizes that simultaneously rotate azimuthally around the ring. The rotation direction and speed depend on the encoded azimuthal phase gradient function and on the analyzer’s rotational speed (see Supplementary Movies). Alternatively, varying the polarization of the illuminating light instead of rotating the analyzer allows for generating different multi-spot patterns in the output.

The compact generation of such polarization-tunable arbitrary complex beams can suggest promising applications in many fields, from life sciences^46–48^ to information^49^ and communication technology^50^.

This versatility in generating customizable structured light fields opens avenues for multiplexing and encoding information, potentially enhancing data transmission rates and channel capacities for high-capacity communication systems^51^. Different patterns with complex azimuthal vector basis configurations and switching between two configurations controlled by the incoming polarized light can represent a method for data signal encoding and transmission. In quantum information systems, generating complex spatial modes with higher-dimensional quantum encoding holds promise for improving the fidelity and capacity of quantum communication networks^52^.

Notably, the ability to generate multiple concentric perfect vortices with azimuthally variant gradients of phase and polarization is interesting for optical trapping. Indeed, the dynamic control in the position and extent of these light regions emerges as an enabling platform for customizable trapping and manipulating of molecules and micro- and nano-particles, enabling precise and dynamic control of the trapped targets movements by acting on the energy flow configuration over time^53,54^. In addition, while the generation of multi-ring patterns can extend the trapping mechanism to low refractive-index targets, the encoding of opposite vectorial nature, *i*.*e*., vortex and antivortex, into two concentric rings offers a sophisticated approach to manipulating the spatial relationships between trapped particles. This novel technique provides unprecedented control, allowing the separation between particles to be increased or, conversely, their interaction to be promoted in a spatially dependent manner, together with the customization of the intensity distribution along the rings^55^. Such versatility opens up new avenues for precise particle manipulation, potentially revolutionizing fields ranging from microfluidics to nanoscale assembly.

Trapping and addressing of atoms and ions for generating entangled qubits on a quantum chip is also an emerging possibility^8^, crucial for realizing integrated quantum computing architectures^56,57^. While cold-controlled interactions among atomic qubits have been observed in microfabricated solid-state devices, this platform lacks efficient and versatile qubit addressability and precise manipulation needed for quantum state engineering. The designed metasurfaces can provide a promising solution to overcome the limitations of the present schemes^58–61^, offering an optical platform compatible with atom trapping devices and providing arbitrary and integrated optical addressing with high precision in polarization, phase, and intensity manipulation, which are essential for qubit excitation, measure, and entanglement control^62^.

For the above reasons, the consolidation of the outlined optical functionalities into compact, flat metaoptics enables an integrated approach that is well-suited for miniaturized beam shaping and light manipulation solutions. Their simplicity, efficiency, and potential for miniaturization position these metaoptics as integral components of next-generation devices, transcending traditional optical systems by providing highly customizable and diverse solutions. The transformative potential demonstrated in this work offers flexible manipulation over a vast set of degrees of freedom for generating complex perfect vortex vector beams, making these metaoptics attractive across a wide range of applications in burgeoning areas of quantum optics, biophotonics, materials science, communication systems, and beyond.

## Methods

### Simulations

Firstly, computational simulations using a tailored finite element method in COMSOL Multiphysics were conducted at a specified operational wavelength of 1310 nm to infer the geometric features of each metaunit. Maintaining the metaatoms matrix periodicity at 600 nm was crucial to comply with the subwavelength regime. Meanwhile, variations in the cross-sectional dimensions of the metaunit were explored within a fixed height of 850 nm. Consequently, metaunit cross sections aligning with the half-wave plate (HWP) condition, represented by ∆ = *π*, were meticulously chosen, where ∆ = *δ*_*x*_ − *δ*_*y*_ signifies the disparity between phase delays for linear polarizations along the *x*− and *y*− axes, respectively. Rigorous criteria were enforced to ensure comprehensive polarization conversion and uniform transmittance across the entire metalens.

Specifically, metaatoms satisfying criteria encompassing a maximum deviation of 0.3 rad from the HWP condition, a transmission difference ∆_*T*_ = *T*_*x*_ − *T*_*y*_ below 0.05, and adhering to a maximum deviation of 0.1 concerning average transmission (*T*_*AVG*_ = (*T*_*x*_ + *T*_*y*_)*/*2) among the simulated metaatoms were selected^63^. Consequently, a library comprising 13 distinct nanopillars, each exhibiting an average transmission of 75%, was established in compliance with the specified prerequisites. Subsequently, given specific phase patterns Φ^±^, the computation of corresponding dynamic and geometric phase maps leads to the required metaatoms configuration for the desired metaoptics. Optical response simulations were conducted using a customized MatLab® code that implements the Fresnel propagator^64^ within a square computational window measuring 600 µm per side, with a pixel size of 600 nm. These simulations were performed on metalenses with a radius of 300 µm, designed for an operational wavelength of 1310 nm, featuring a 13-level discretization of dynamic phase, and illuminated by a Gaussian beam with a 150 µm waist radius.

### Fabrication

**Fig. 5:**
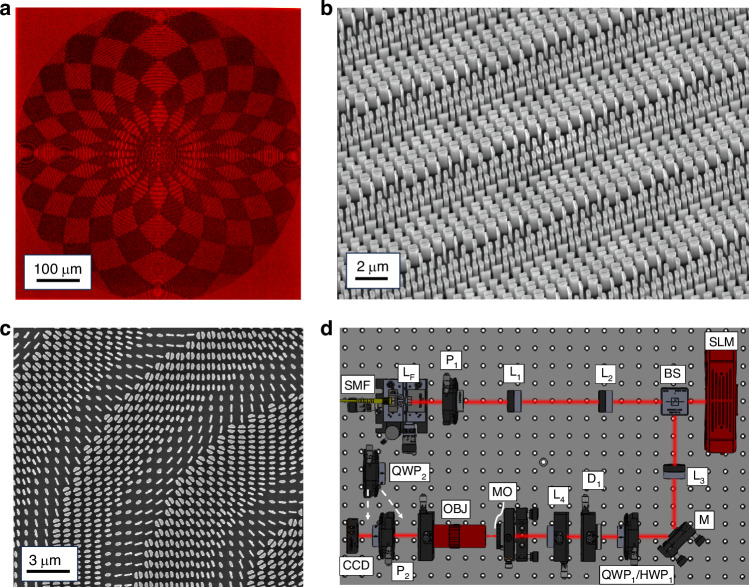
Fabricated samples and experimental setup. **a** Brightfield microscope image of the metaoptics in Fig. 3. **b**, **c** are tilted and top-view SEM inspections of the sample whose response is depicted in Fig. 4c–e, respectively. **d** Optical layout for vector beam characterization. *SMF* single mode fiber of a DFB laser; L_F_ aspheric lens with focal length *f*_*F*_ = 7.5 mm, *P*_1_ linear polarizer; telescope system L_1_ and L_2_ with *f*_1_ = 3.5 cm and *f*_2_ = 10.0 cm; *BS* non-polarized beam splitter; *SLM* LCoS spatial light modulator (SLM), *M* mirror; *QWP*_1_ quarter-wave plate, *HWP*_1_ half-wave plate, 4-*f* system (lenses L_3_ and L_4_) with *f*_3_ = 20.0 cm and *f*_4_ = 12.5 cm, *D*_1_ iris diaphragm, *MO* metaoptics, *Obj* 20× objective, *QWP*_2_ quarter-wave plate, *P*_2_ linear polarizer, *CCD* charge-coupled device camera

The metaoptics under examination were fabricated employing a two-stage fabrication procedure. Electron beam lithography (EBL) was initially used to transpose the optimized computational blueprint onto the physical sample^65^. This process entailed patterning a thin PMMA resist layer on $\langle 100\rangle$ silicon substrates through an EBL system (Carl Zeiss Sigma 300, 30 keV beam voltage). Subsequently, an alumina mask was applied via electron gun evaporation of an *Al*_2_*O*_3_ target within high vacuum conditions. Following the development stage, an alumina layer remained on the previously exposed regions after a lift-off process conducted through sonication in hot acetone. Ultimately, the pattern was transferred onto the underlying silicon substrate via Inductively Coupled Plasma Reactive Ion Etching (ICP-RIE). All fabricated metasurfaces maintained a consistent diameter of 600 µm. Figure 5a–c depicts the morphological characterization of a sample.

### Optical setup

The optical performance assessment of the metaoptics was conducted using the experimental arrangement detailed in Fig. 5d. The output from a DFB laser (*λ* = 1310 nm, 1310LD34 1-2-2-1 CCSI, AeroDiode) was collimated at the termination of a single mode fiber using an aspheric lens with a focal length of *f*_*F*_ = 7.5 mm (A375TM-C, Thorlabs). Initially linearly polarized, the beam underwent expansion via an initial telescope system (*f*_1_ = 3.5 cm, *f*_2_ = 10.0 cm) before illuminating the display of an LCoS (Liquid Crystal on Silicon) spatial light modulator (X13267-08, Hamamatsu, pixel pitch 12.5 µm). We generated the desired input Gaussian beam employing a phase and amplitude modulation technique^66^. Utilizing a 4-*f* system (*f*_3_ = 20.0 cm, *f*_4_ = 12.5 cm) featuring an aperture in the Fourier plane facilitated both the isolation of the first-order encoded mode and the adjustment of the beam waist to *W* = 150 µm. A 50:50 beam-splitter positioned before the SLM directed the reflected beam along the desired optical pathway. Within this path, a quarter-wave plate (QWP_1_) (WPQ10M1310, Thorlabs) or a half-wave plate (HWP_1_ in Fig. 5d) (WPH05M-1310, Thorlabs) were employed to establish the desired polarization state. Specifically, the QWP transformed the beam from horizontally polarized to circularly polarized states. At the same time, the HWP alternatively rotated the polarization plane to generate the desired vector beam upon exiting the SLM (see Supplementary Material). The polarized beam was resized accordingly to illuminate the patterned zone of the optics fixed on a 6-axis kinematic mount (K6XS, Thorlabs). The collection of the output beam involved a long working distance 20x Objective (MY20X-824 - Plan Apochromat Objective, 480–1800 nm, 0.40 NA, 20.0 mm WD, Mitutoyo) mounted on a micrometric translator stage (LX20/M, Thorlabs). Employing removable additional linear polarizer (P_2_) and quarter-wave plate (QWP_2_) enabled the analysis of the VB configuration and the generation of the Stokes polarimetric projections, respectively. Image capture of the collected beam was made by using a camera (WiDy SWIR 640U-S, pixel pitch 15 µm). The discrepancies in scale bars between simulations and experimental acquisitions in the figures were due to the introduced magnification factor. Proper extraction and magnification of generated beams necessitated a microscope objective, as the 1.5 mm focal length was inadequate for direct collection on the CCD camera. Moreover, optimal appreciation of the beam exploited the entire sensitive area of the camera. Finally, the experimental efficiency of the metaoptics was quantified, yielding an average transmission value of 72%, closely approximating the anticipated theoretical value of 75%.

## Supplementary information

Supplementary Material

Video 1

Video 2

Video 3

Video 4

Video 5

Video 6

Video 7

## Data Availability

The datasets generated during and/or analyzed during the current study are available from the corresponding author upon reasonable request.
