# Supplementary material for: Azimuthally-variant perfect vector beams for the control of arbitrary phase and polarization ring patterns
Source: Light Sci Appl. 2025 May 6;14:183. doi: 10.1038/s41377-025-01859-1 (PMC12053751; doi:10.1038/s41377-025-01859-1)
Supplement: Supplementary file 1 — Supplementary Material [file 41377_2025_1859_MOESM1_ESM.pdf]

Supplementary Information for Azimuthally-variant perfect vector beams for the control of arbitrary phase and polarization ring patterns

Andrea Vogliardi[1,2*], Gianluca Ruffato[1,2,3*], Daniele Bonaldo[2,3], Simone Dal Zilio[4], and Filippo Romanato[1,2,4]

[1]Department of Physics and Astronomy 'G. Galilei', University of Padova, Via Marzolo 8, Padova, 15131, Italy.
[2]Padua Quantum Technology Center 'QTech', University of Padova, Via Gradenigo 6, Padova, 15131, Italy.
[3]Department of Information Engineering, University of Padova, Via Gradenigo 6, Padova, 15131, Italy.
[4]Istituto Officina dei Materiali, CNR, S.S. 14 - Km. 163,5, Trieste, 34149, Italy.

*Corresponding author(s). E-mail(s): andrea.vogliardi@phd.unipd.it; gianluca.ruffato@unipd.it;
Contributing authors: daniele.bonaldo2@phd.unipd.it; dalzilio@cnr.iom.it; filippo.romanato@unipd.it;

S1 Generation of Vector beams using dual-functional metalenses

The mathematical description of vector beams involves introducing a 4-dimensional basis, formed by the Cartesian product of a 2D spatial mode basis (with states $|+\ell\rangle$ and $|-\ell\rangle$) and a 2D polarization basis (with states $|R\rangle$ and $|L\rangle$). Here, $|R\rangle = [1\ i]^T$ and $|L\rangle = [1\ -i]^T$ represent right- and left-handed circular polarizations, respectively, with the normalization factor $1/\sqrt{2}$ omitted. The states $|\pm\ell\rangle$ correspond to ℓ-th order Orbital Angular Momentum (OAM) modes.

The 4D space can be formally represented as the direct sum of two distinct 2D subspaces known as Hybrid Poincaré Spheres (HPSs) [1]. These HPSs encompass the vortex states, characterized by the basis $\{u_R^+, u_L^+\}$, and the anti-vortex states, defined by the basis $\{u_R^-, u_L^-\}$:

$$\{|+\ell\rangle, |-\ell\rangle\} \otimes \{|R\rangle, |L\rangle\} = \{u_R^+, u_L^+\} \oplus \{u_R^-, u_L^-\} \tag{1}$$

Subsequently, the comprehensive state of an ℓ-th order vector beam can be expressed in the following terms, involving Orbital Angular Momentum (OAM) beams and circular polarization states:

$$W^{\pm}_{\vartheta,\chi} = cos(\chi)e^{-i\vartheta}|\pm\ell\rangle|L\rangle + sin(\chi)e^{+i\vartheta}|\mp\ell\rangle|R\rangle \tag{2}$$

where the two angles, denoted as ϑ and χ, correspond to the coordinates of the associated point on the Hybrid Poincaré Sphere (HPS).

To implement these properties in light, spin-decoupled dual-functional metalenses (DFMLs) can be used, independently managing two incident beams with opposite circular polarizations (left- and right-handed). This is achieved by carefully selecting the nanopillar shape and rotation angle, allowing simultaneous control of both dynamic and geometric phases. The action of a metaunit on the circular polarization basis $\{|R\rangle, |L\rangle\}$ is described by the matrix J:

$$\begin{aligned} J|L\rangle &= -ie^{i(\delta_x+\delta_y)/2}e^{+i2\theta}|R\rangle = -ie^{i\Phi_{dyn}}e^{+i\Phi_{geo}}|R\rangle = e^{i\Phi^+}|R\rangle \\ J|R\rangle &= -ie^{i(\delta_x+\delta_y)/2}e^{-i2\theta}|L\rangle = -ie^{i\Phi_{dyn}}e^{-i\Phi_{geo}}|L\rangle = e^{i\Phi^-}|L\rangle \end{aligned} \tag{3}$$

where δ_x and δ_y refer to the local phase delay that a metaunit must impart to TM and TE linear polarizations respectively arising a polarization-insensitive dynamic phase, and 2θ is the polarization-sensitive geometric phase arising from a nanopillar's rotation of θ relative to the x-axis. This holds when the half-wave condition is met ($\delta_y = \delta_x + \pi$) and the transmission amplitudes are equal in both directions [2].

Since a linearly polarized state can be written as a superposition of circular polarization components $|\vartheta\rangle = \frac{|R\rangle e^{i\vartheta} + |L\rangle e^{-i\vartheta}}{\sqrt{2}}$, it is possible to control the wavefronts of

both circularly polarized components simultaneously:

$$J|\vartheta\rangle = \frac{e^{i\vartheta}e^{i^{-}}|L\rangle + e^{-i\vartheta}e^{i^{+}}|R\rangle}{\sqrt{2}} \tag{4}$$

This enables the creation of a non-separable combination of polarization and spatial modes, as indicated in Equation 2 [2].

To achieve the desired phase patterns $\Phi^{\pm}$ using a dual-functional metaoptics, the formulas in Eq. 3 have to be reversed finding the relationship between phase delay and metaatom features:

$$\begin{aligned} \delta_x &= \frac{\Phi^{+} + \Phi^{-}}{2} \\ \delta_y &= \frac{\Phi^{+} + \Phi^{-}}{2} + \pi \\ \theta &= \frac{\Phi^{+} - \Phi^{-}}{4} \end{aligned} \tag{5}$$

allowing also the formal evaluation of the dynamic and geometric phase from the desired spin-decoupled phase delays:

$$\begin{aligned} \Phi_{dyn} &= \frac{\Phi^{+} + \Phi^{-}}{2} + \frac{\pi}{2} \\ \Phi_{geo} &= \frac{\Phi^{+} - \Phi^{-}}{2} \end{aligned} \tag{6}$$

S2 Orbital angular momentum carried by azimuthally-variant vortex beams

To evaluate the orbital angular momentum carried by our vortex beams, we calculated the topological charge (Q) by integrating the beam phase along a closed path around the central singularity [3]

$$Q = \frac{1}{2\pi}\oint d\chi = \frac{1}{2\pi}\oint d\mathbf{s}\cdot\nabla\chi \tag{7}$$

where d**s** is the line element, χ is the phase of the beam. Substituting the phase of the beam with the one we transferred through the metaoptics, taking into account that the axicon and lens term have circular symmetry and changing the integration variable, we can derive:

$$\begin{aligned} Q &= \frac{1}{2\pi}\oint d\mathbf{s}\cdot\nabla\Phi^{\pm} = \frac{1}{2\pi}\oint d\mathbf{s}\cdot\nabla\Phi_{m,\ell_0,\ell_1} = \\ &= \frac{1}{2\pi}\oint d\varphi\cdot\nabla\left(\sum_{j=1}^{m}(-1)^{H_C^j}[\pm(\ell_0 + H_G^j\ell_1) \pm (-1)^{H_G^j}(\ell_1-\ell_0)\beta(m\cdot\varphi)]\varphi\right) \end{aligned} \tag{8}$$

where only Φ_{m,ℓ_0,ℓ_1} plays a role in calculating the topological charge. In the simplest case where $m = 1$, $H_C^1 = H_G^1 = 0$ and $\beta(m\cdot\varphi) = C$ (constant), it is trivial to obtain $Q = C(\ell_1-\ell_0)-\ell_0$, with an associated orbital angular momentum of $\hbar Q$. In this case, the topological charge can be visualized as the number of $0-2\pi$ phase jumps [4], and the helicity depends on the gradient sign. When $\beta(m\cdot\varphi)$ is not a constant and $m > 1$ the solution is not trivial and leads to interesting cases such as generating vector beams from two beams in orthogonal polarization states but with no topological charge. To intuitively evaluate this behaviour, we can think of an azimuthally variant perfect vortex divided into two sectors ($m = 2$) with one of these two sectors having the inversion of topological charge (i.e., $H_C^1 = 0$ and $H_C^2 = 1$) but no inversion of phase gradient (i.e., $H_G^1 = H_G^2 = 0$). In this configuration, without any dependence from the gradient function β, half of the ring has a positive (negative) phase gradient from $\pm\ell_0$ to $\pm\ell_1$; instead, the other half has a negative (positive) phase gradient from $\mp\ell_0$ to $\mp\ell_1$. Thus, we have exactly the same number of phase jumps from 0 to 2π but opposite helicity in the two sectors, leading to a null topological charge. In a mathematical form, it can be expressed as:

$$\begin{aligned} Q &= \frac{1}{2\pi}\oint d\varphi\cdot\nabla\left(\sum_{j=1}^{2}(-1)^{H_C^j}[\pm\ell_0 \pm (\ell_1-\ell_0)\beta(m\cdot\varphi)]\varphi\right) = \\ &= \frac{1}{2\pi}\left(\int_0^{\pi} d\varphi\cdot\nabla[\pm\ell_0 \pm (\ell_1-\ell_0)\beta(m\cdot\varphi)]\varphi\right) + \\ &+ \frac{1}{2\pi}\left(\int_{\pi}^{2\pi} d\varphi\cdot\nabla[\mp\ell_0 \mp (\ell_1-\ell_0)\beta(m\cdot\varphi)]\varphi\right) = 0 \end{aligned} \tag{9}$$

Now, implementing two azimuthally variant perfect vortices following the dual functional paradigm for vector beams, we will obtain (under the illumination of linearly polarized light) the generation of two rings, respectively LCP and RCP polarized, carrying a null topological charge but with opposite azimuthal phase values (Eq. 4). It is worth noting that the same result is achievable if we enabled the azimuthal phase gradient inversion rather than the topological charge inversion in a single sector. This can be easily explained by looking at the 0-2π phase jumps. Performing the phase gradient inversion in one of the two sectors, the number of 0-2π phase jumps in both sectors but with opposite helicity, leading to an overall null topological charge. Such behaviour is reported and verified from the optical response of metaoptics depicted in Fig. S1, Fig. 3.

S3 Metaoptics design parameters

Ref.	$\beta(\cdot)$	N	α	m	H_C	H_G	ℓ_0	ℓ_1
Fig. 2	$\frac{\varphi}{2\pi}$	2	$\alpha_{in} = 0.38$	$m_{in} = 1$	$H^1_{C_{in}} = 0$	$H^1_{G_{in}} = 0$	$\ell_{0_{in}} = 0$	$\ell_{1_{in}} = 7$
			$\alpha_{out} = 0.43$	$m_{out} = 1$	$H^1_{C_{out}} = 1$	$H^1_{G_{out}} = 0$	$\ell_{0_{out}} = 0$	$\ell_{1_{out}} = 7$
Fig. 3	$\frac{\varphi}{2\pi}$	2	$\alpha_{in} = 0.38$	$m_{in} = 4$	$H^1_{C_{in}} = 0$	$H^1_{G_{in}} = 1$	$\ell_{0_{in}} = 5$	$\ell_{1_{in}} = 10$
					$H^2_{C_{in}} = 1$	$H^2_{G_{in}} = 0$		
					$H^3_{C_{in}} = 0$	$H^3_{G_{in}} = 1$		
					$H^4_{C_{in}} = 1$	$H^4_{G_{in}} = 0$		
			$\alpha_{out} = 0.43$	$m_{out} = 4$	$H^1_{C_{out}} = 1$	$H^1_{G_{out}} = 1$	$\ell_{0_{out}} = 5$	$\ell_{1_{out}} = 10$
					$H^2_{C_{out}} = 0$	$H^2_{G_{out}} = 0$		
					$H^3_{C_{out}} = 1$	$H^3_{G_{out}} = 1$		
					$H^4_{C_{out}} = 0$	$H^4_{G_{out}} = 0$		
Fig.4	$\frac{\exp(A \cdot \frac{\varphi}{2\pi})}{\exp(A)}$	1	$\alpha = 0.16$	$m = 1$	$H_C = 0$	$H_G = 0$	$\ell_0 = 0$	$\ell_1 = 3$
Fig.4	$\frac{sin\varphi}{\pi\varphi}$	1	$\alpha = 0.16$	$m = 1$	$H_C = 0$	$H_G = 0$	$\ell_0 = 1$	$\ell_1 = 3$
Fig. S1	$\frac{\varphi}{2\pi}$	2	$\alpha_{in} = 0.38$	$m_{in} = 2$	$H^1_{C_{in}} = 0$	$H^1_{G_{in}} = 0$	$\ell_{0_{in}} = 0$	$\ell_{1_{in}} = 5$
					$H^2_{C_{in}} = 0$	$H^2_{G_{in}} = 0$		
			$\alpha_{out} = 0.43$	$m_{out} = 2$	$H^1_{C_{out}} = 0$	$H^1_{G_{out}} = 0$	$\ell_{0_{out}} = 0$	$\ell_{1_{out}} = 5$
					$H^2_{C_{out}} = 0$	$H^2_{G_{out}} = 0$		
Fig. S1	$\frac{\varphi}{2\pi}$	2	$\alpha_{in} = 0.38$	$m_{in} = 2$	$H^1_{C_{in}} = 0$	$H^1_{G_{in}} = 0$	$\ell_{0_{in}} = 0$	$\ell_{1_{in}} = 5$
					$H^2_{C_{in}} = 0$	$H^2_{G_{in}} = 1$		
			$\alpha_{out} = 0.43$	$m_{out} = 2$	$H^1_{C_{out}} = 1$	$H^1_{G_{out}} = 0$	$\ell_{0_{out}} = 0$	$\ell_{1_{out}} = 5$
					$H^2_{C_{out}} = 1$	$H^2_{G_{out}} = 1$		
Fig. S1	$\frac{\varphi}{2\pi}$	2	$\alpha_{in} = 0.38$	$m_{in} = 2$	$H^1_{C_{in}} = 0$	$H^1_{G_{in}} = 1$	$\ell_{0_{in}} = 5$	$\ell_{1_{in}} = 10$
					$H^2_{C_{in}} = 0$	$H^2_{G_{in}} = 0$		
			$\alpha_{out} = 0.43$	$m_{out} = 2$	$H^1_{C_{out}} = 1$	$H^1_{G_{out}} = 1$	$\ell_{0_{out}} = 5$	$\ell_{1_{out}} = 10$
					$H^2_{C_{out}} = 1$	$H^2_{G_{out}} = 0$		
Fig. S5	$\frac{\varphi}{2\pi}$	3	$\alpha_{in} = 0.50$	$m_{in} = 2$	$H^1_{C_{in}} = 0$	$H^1_{G_{in}} = 0$	$\ell_{0_{in}} = 0$	$\ell_{1_{in}} = 3$
					$H^2_{C_{in}} = 1$	$H^2_{G_{in}} = 0$		
			$\alpha_{mid} = 0.58$	$m_{mid} = 1$	$H^1_{C_{mid}} = 0$	$H^1_{G_{mid}} = 0$	$\ell_{0_{mid}} = 0$	$\ell_{1_{mid}} = 5$
			$\alpha_{out} = 0.66$	$m_{out} = 2$	$H^1_{C_{out}} = 0$	$H^1_{G_{out}} = 0$	$\ell_{0_{out}} = 0$	$\ell_{1_{out}} = 7$
					$H^2_{C_{out}} = 1$	$H^2_{G_{out}} = 0$		

Table 1 Parameters encoded in manuscript's Eq. 4 to generate each metaoptics reported in the work. For the benefit of the reader, the first column indicates the reference related to each optics.

S4 Metaoptics generating two rings AV-PVBs

We designed three metaoptics to evaluate the effect of each design parameter on the target beam. Firstly, we imposed 0 as background charge and 5 as gradient charge. In both sectors, the inner and outer rings were in the vortex state. In this case, we didn't enable the inversion of the phase gradient in any sector. In the first row of Figure S1, we compare the second optics' simulation and experimental characterization. It can be observed that the sectors' designs dictate azimuthal variation. Additionally, the inner and outer ring lobes rotate in the same direction as the rotating analyzer, confirming that the two rings are in the same vectorial state. In the second optics, we add the inversion of the phase gradient in the second sector of both rings. The inner ring is in the vortex state in both sectors, and the outer one is in the anti-vortex state. Here, the overall topological charge of both rings for the orthogonal LCP and RCP constituent components is 0, as we have opposite phase gradients in the two sectors of each ring. This generates vector beams from the non-separable combination of spin and non-charged beams. In the second row of Figure S1, we compare the third optics' simulation and experimental characterization. The inner and outer ring lobes rotate in opposite directions, confirming that the two rings are in opposite vectorial states. We designed the last optics to show that having $\ell_{1_j} = 0$ is unnecessary, but the starting topological charge can be chosen arbitrarily. We divided the two rings into two sectors; we imposed 5 as the background charge and 10 as the gradient charge. We added the inversion of the phase gradient in the first sector of both rings, and finally, we forced the inner ring into the vortex state and the outer one into the anti-vortex state. Also, in this case the topological charge of the LCP and RCP components is equal to zero. The inner and outer ring lobes rotate in opposite directions, confirming that the two rings are in opposite vectorial states. In all three cases, both rings exhibit $2\ell_1$ spots with different sizes, confirming the azimuthally-variant behaviour after the filtering.

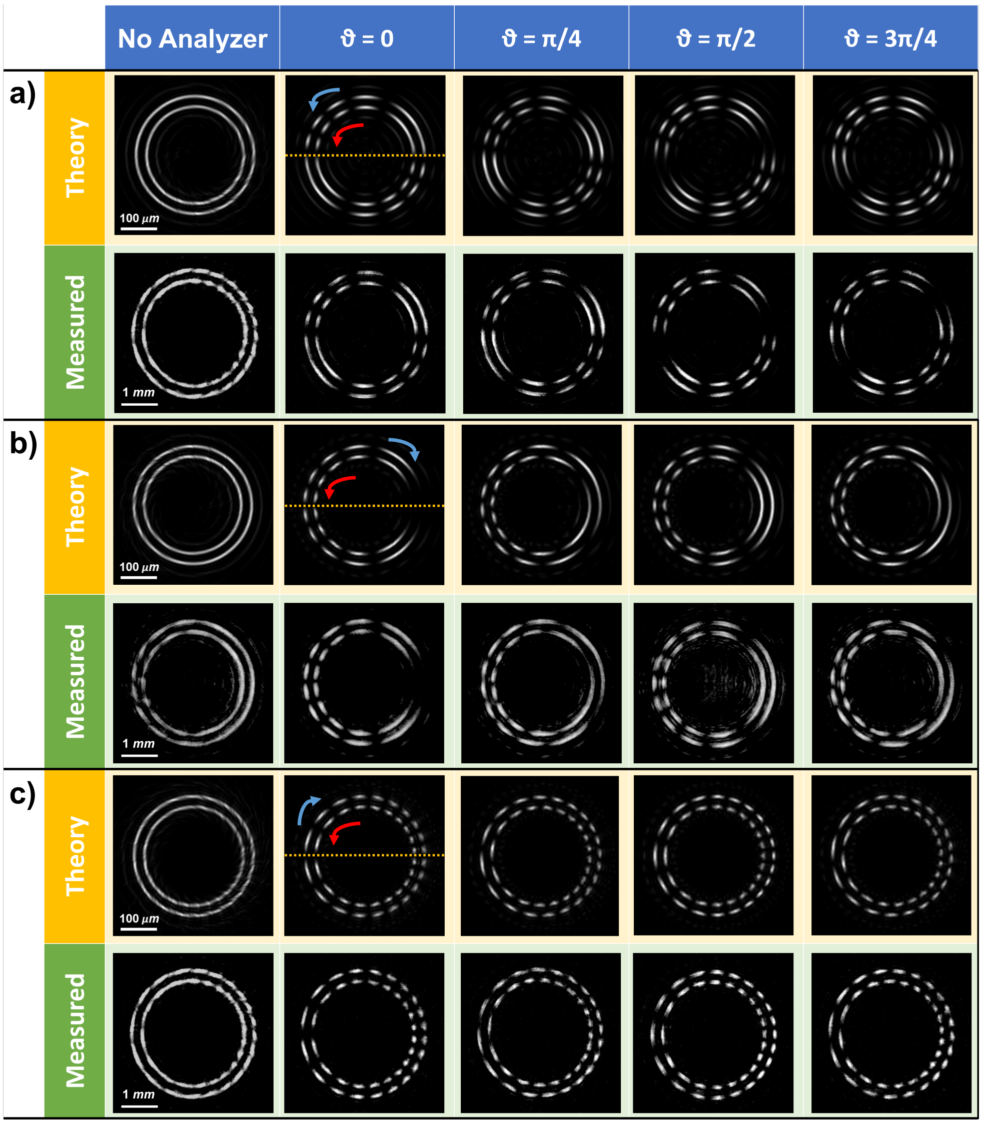

Fig. S1 Simulated and measured vector beams' intensity pattern after filtering with a linear polarizer. In the first case a), the two rings are made by two constituent sectors that rotate in the same direction because no different topological charge inversion has been enabled in sectors of the same ring. On the other hand b)-c), the two rings can rotate in opposite directions depending on which state has been encoded. As expected, the background charge and the gradient charge (ℓ_0 and ℓ_1, respectively) affect the number and the size of the spots. The measured patterns fit well with the simulated one, as confirmed by the profile analysis.

S5 Simulated and Experimental spots size and rotation

For completeness, we reported the simulated and experimental profiles of the filtered VBs for all the fabricated metaoptics. The experimental profiles follow the theoretical ones for both the rings, for any rotation of the analyzer for each of the fabricated metaoptics (Fig. S2-S4).

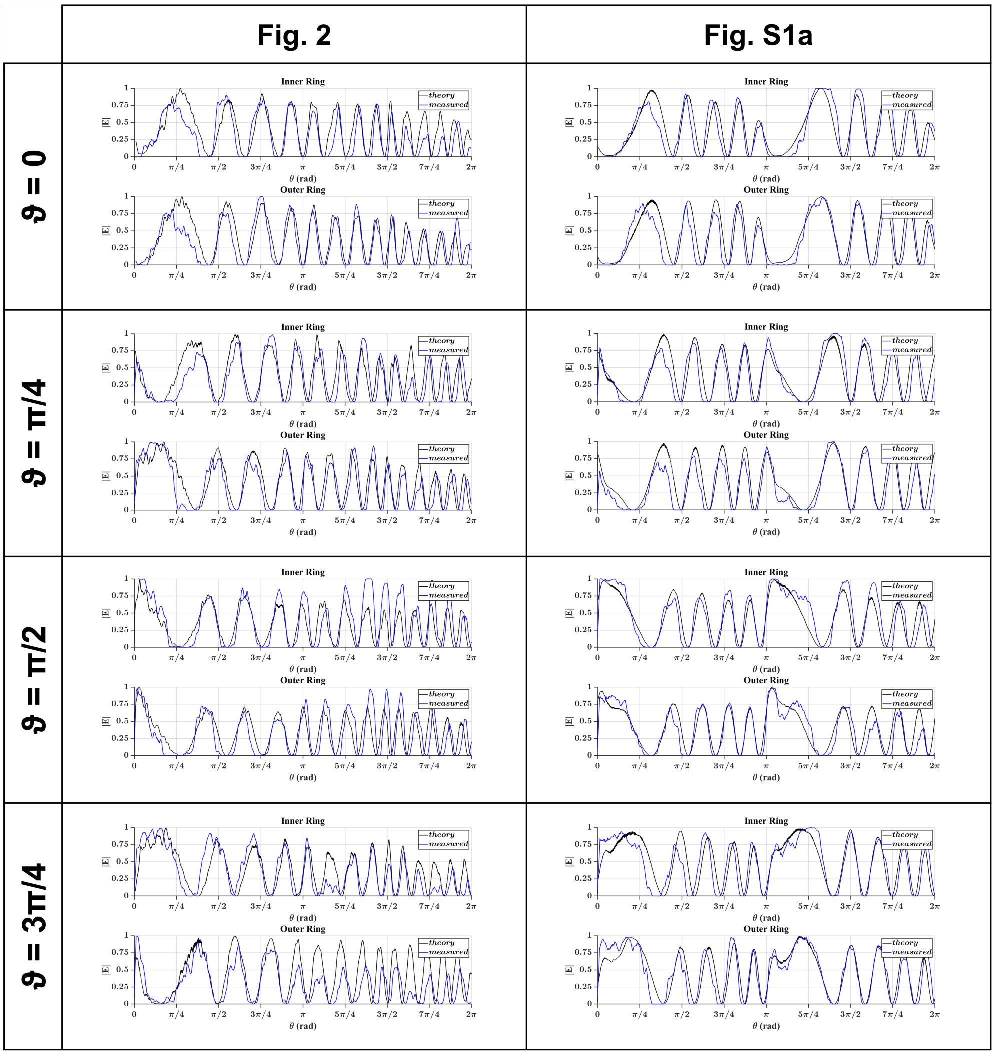

Fig. S2 Comparison between the theoretical (simulated) and measured azimuthal intensity profiles of the filtered vector beams with different rotations of the analyzer for both the inner and the outer ring. Plots refer to metaoptics reported in Fig.2 and Fig S1a.

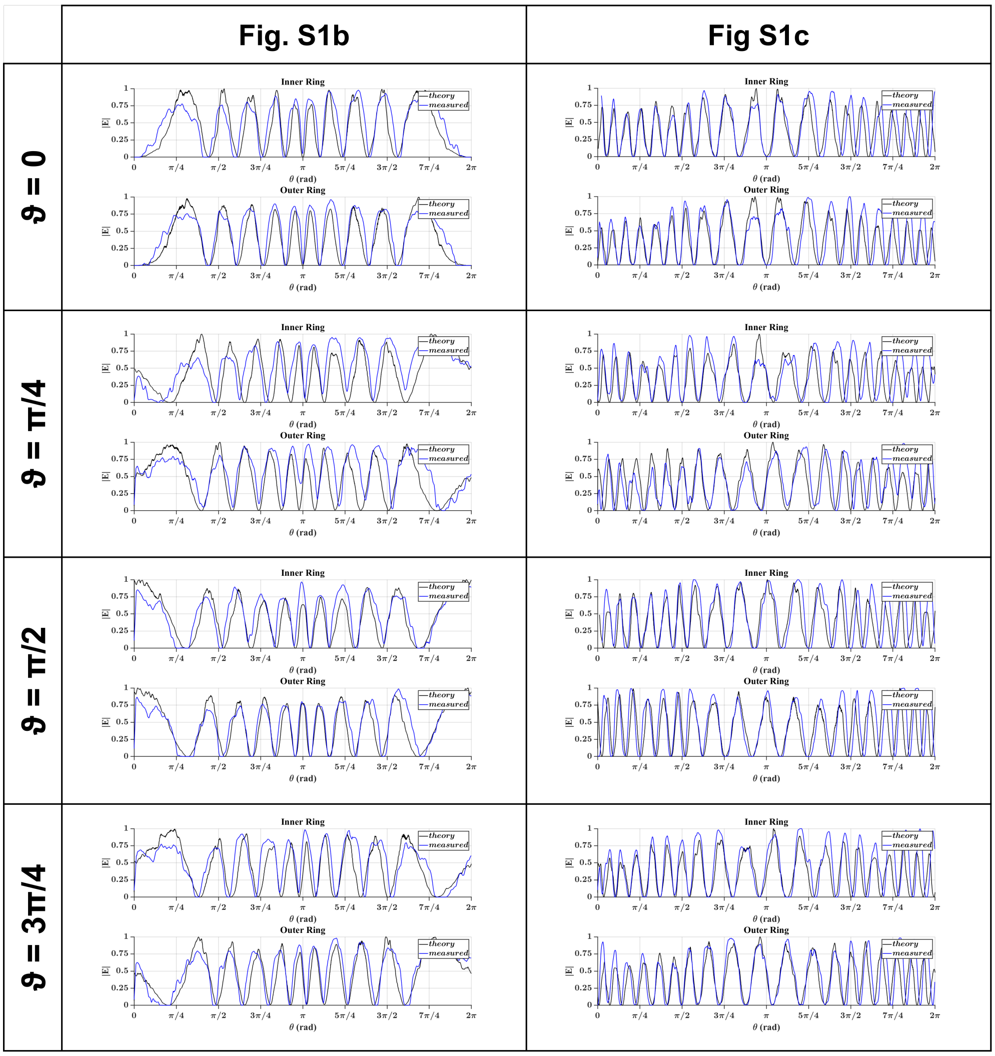

Fig. S3 Comparison between the theoretical (simulated) and measured azimuthal intensity profiles of the filtered vector beams with different rotations of the analyzer for both the inner and the outer ring. Plots refer to metaoptics reported in Fig.S2b-c.

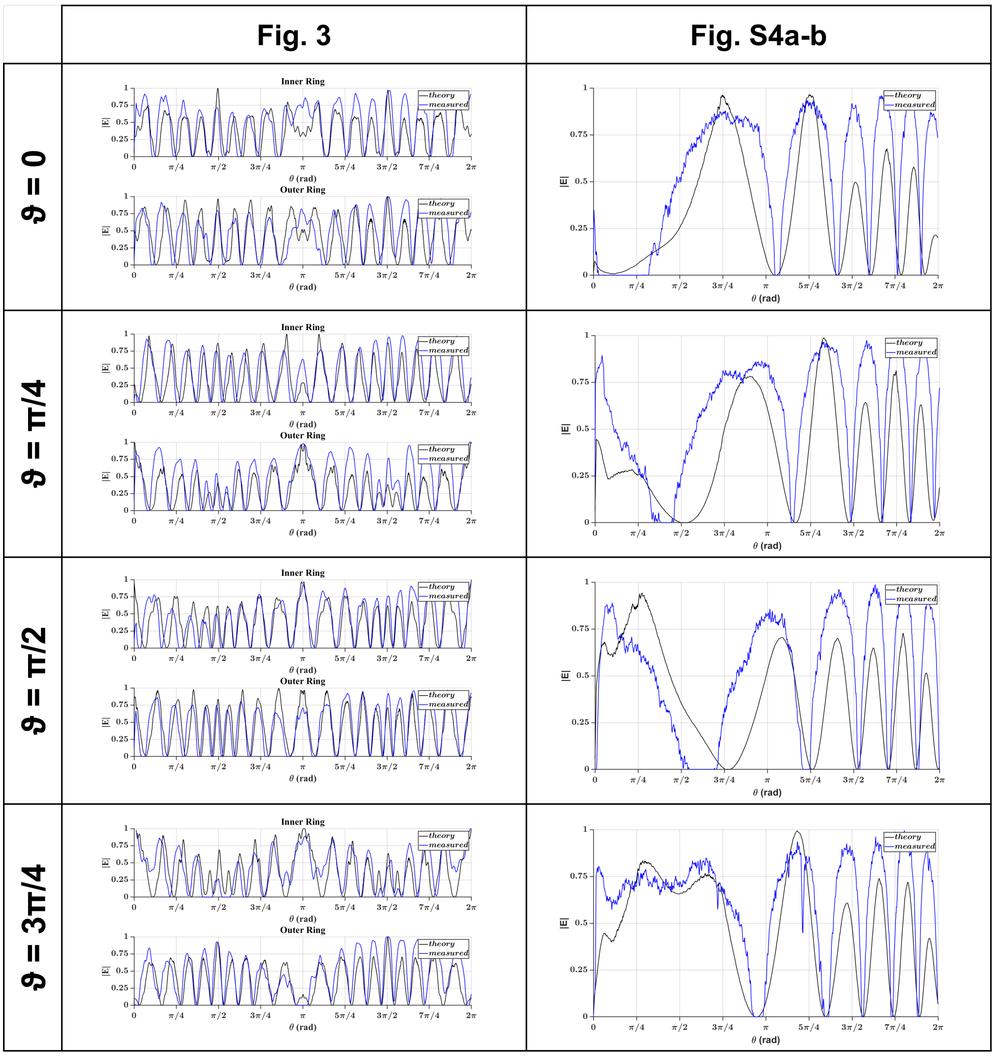

Fig. S4 Comparison between the theoretical (simulated) and measured azimuthal intensity profiles of the filtered vector beams with different rotations of the analyzer for the inner and the outer ring in the first column, and single ring in the second column. Plots refer to metaoptics reported in Fig.3 and Fig. 4a-b.

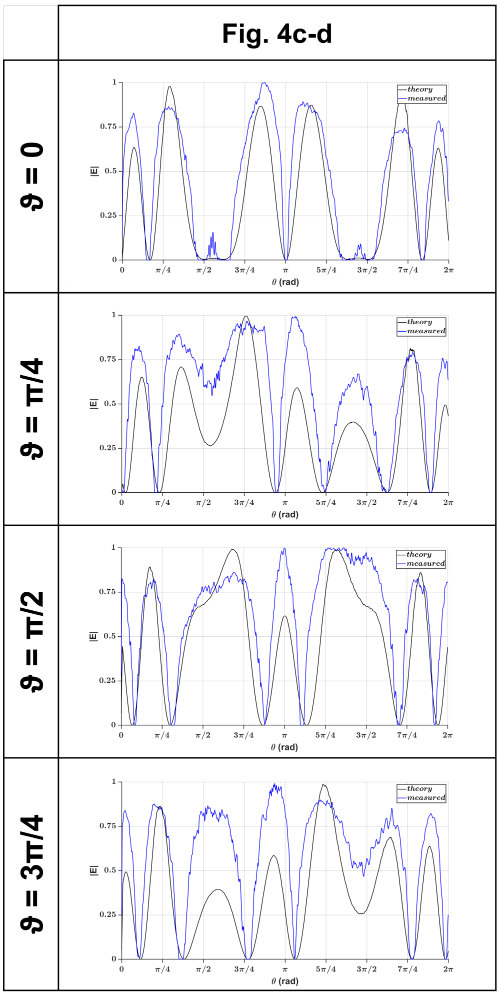

Fig. S5 Comparison between the theoretical (simulated) and measured azimuthal intensity profiles of the filtered vector beams with different rotations of the analyzer. Plots refer to metaoptics reported in Fig. 4c-d.

S6 Multiple concentric rings with different vortex states

In this section, we simulated the behaviour of metaoptics encoding more complicated multi-ring AV-PVBs. The simulated metaoptic generates three concentric rings divided into different sectors: $m_{in} = 2$, $m_{mid} = 1$ and $m_{out} = 2$. Each concentric ring carries an azimuthally-variant topological charge; we impose 0 as background topological charge and the gradient charges of 3,5 and 7, respectively. Regarding the vectorial state, we impose to the inner ring the vortex state in the first sector and the anti-vortex state in the second. For the central ring, we set the vortex state; otherwise, the outer one has the same vectorial configuration as the inner one. We don't enable the local phase gradient inversion in this case.

We show the simulative results in Figure S6. We can see that three scalar rings carrying an azimuthally-variant gradient have been generated under the illumination of circularly polarised light. (Fig. S6 c-d) Moreover, depending on the handedness of the polarization, the three rings exhibit a positive or negative phase gradient. In particular, the central ring exhibits an opposite phase gradient since the anti-vortex state has been encoded. Figure S6 e-f shows the variation of the electric field's amplitude components (E_x and E_y) along the ring and the polarization plot of the generated beam at the focal plane under the illumination of a vertically polarized light. In particular, we can observe the continuous azimuthal variation of the polarization and the $|2\ell_{1,i}|$ jumps between vertical and horizontal polarization that confirm the beam vectorial state. Finally, in Figure S6 g, we show the simulations of the analyzed vector beam, simulating a rotating linear polarizer as an analyzer to capture the vectorial nature of the beams. After the filtering, all the rings exhibit $|2\ell_1|$ spots of different sizes, which confirms both the azimuthally-variant and the vectorial nature of the beam. As expected, the spots belonging to a sector in the vortex state rotate in the same direction as the rotating analyzer. Otherwise, the spots belonging to a sector in the anti-vortex state rotate in the counter direction, confirming that the sectors are in opposite vectorial states.

Finally, we simulated the optical response under horizontally polarized light. As described in the manuscript, changing the impinging light's polarization makes generating the orthogonal polarization pattern possible. We confirmed this behaviour through filtering analysis, as depicted in Figure S6g.

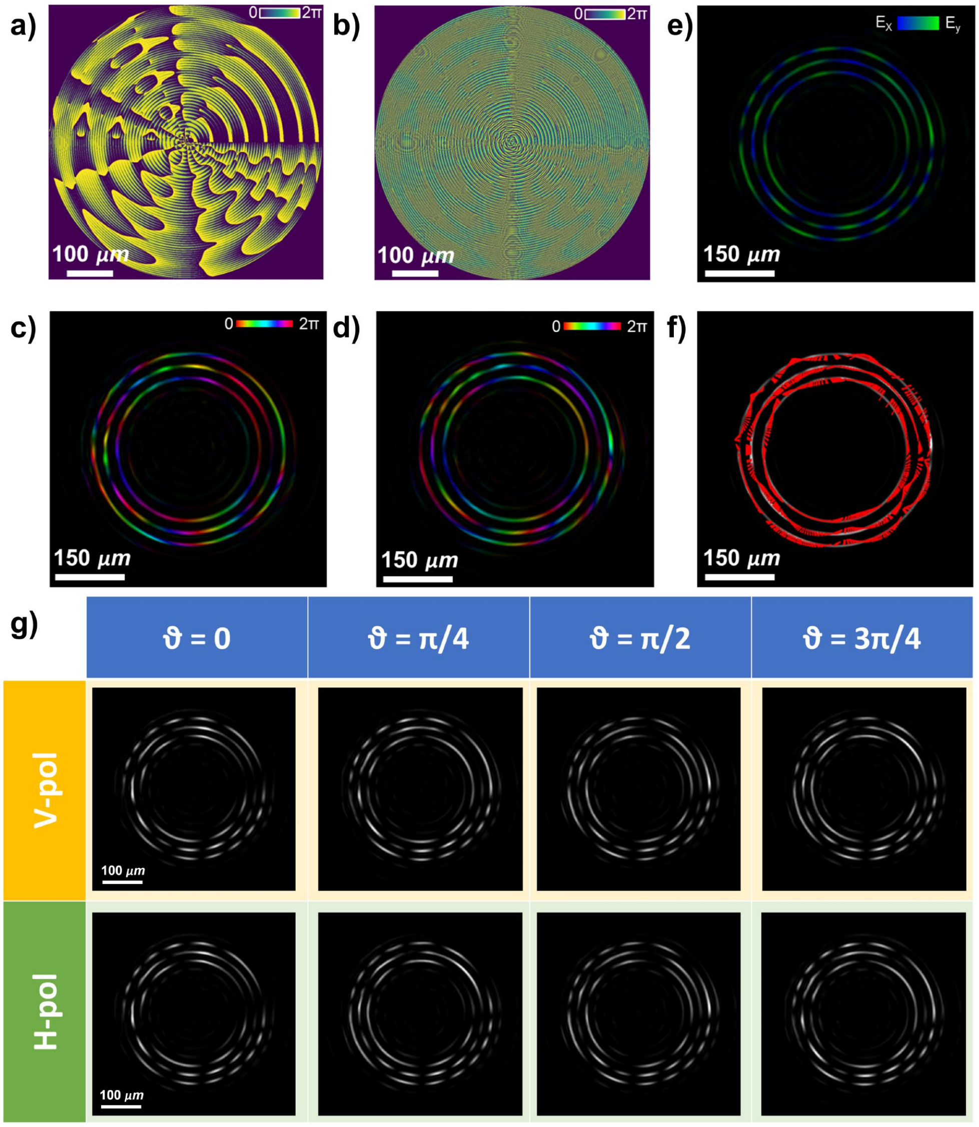

Fig. S6 a) and b) show the encoded dynamic and geometric phases, respectively, of the designed metaoptics. Colours refer to the phase range from 0 to 2π. c) and d) depicts the simulated LCP and RCP optical response of the metaoptics under the illumination of a Gaussian beam. Brightness and colours refer to intensity and phase. e) X- and Y- components of the electric field and f) polarization pattern of the azimuthally-variant perfect vector beam generated under the illumination of a vertically polarized Gaussian beam. g) simulated and measured intensity pattern after filtering the vector beam with a linear polarizer. The bright spots appear in opposite configurations depending on the impinging polarization, confirming the orthogonality between the two polarization patterns. The different local azimuthal variation of phase gradient that induces different spot sizes is appreciable in all three rings.

S7 Generation of hybrid-order azimuthally-variant vector beams

In an extended scenario, we can break the balancing of topological charges between the two constituent LCP and RCP beams by illuminating the dual-functional metaoptics that generate VBs with a beam that is itself topologically charged (such as a Laguerre-Gaussian beam or a Perfect Vortex Beam). In this way, it is possible to generate hybrid-order azimuthally-variant vector beams. Such behaviour is explained by the fact that such metalenses, when illuminated with a topologically charged beam, act as charge adders for the LCP and RCP phase response [5]. Thus, we can rewrite the equation of a ℓ order vector beam under the illumination of a charged beam with topological charge Q:

$$W^{\pm}_{\vartheta,\chi} = cos(\chi)e^{-i\vartheta}|\pm\ell + Q\rangle|L\rangle + sin(\chi)e^{+i\vartheta}|\mp\ell + Q\rangle|R\rangle \tag{10}$$

where, due to linearity, the illuminating TC is equally distributed in the LCP and RCP components. For both the vortex and anti-vortex states, the polarization pattern of the vector beam order is still the same as the sum of the absolute values of the topological charges carried by the two constituent polarizations doesn't change:

$$W^{\pm}_{order} = |\pm\ell + Q| - |\mp\ell + Q| = 2|\ell| \tag{11}$$

On the other hand, the overall topological charge is equal to the topological charge carried by the impinging beam:

$$W^{\pm}_{TC} = \frac{(\pm\ell + Q) + (\mp\ell + Q)}{2} = Q \tag{12}$$

We report simulations of the optical response of a metaoptics generating unbalanced AV-PVB divided in one sector ($m = 1$) with no topological charge or phase gradient inversion ($H^1_C = H^1_G$), $\ell_0 = 0$ and $\ell_1 = 5$, under the illumination of charged light. As shown in Figure S7, the two constituent circularly polarized components are unbalanced if the beam is illuminated with a beam carrying topological charge. Thus, under the illumination of a linearly polarized charged beam, on the one hand, the TC transferred to the AV-PVB is the same as the impinging one, on the other hand, the polarization pattern is fixed and it is strictly dependent on the design parameters.

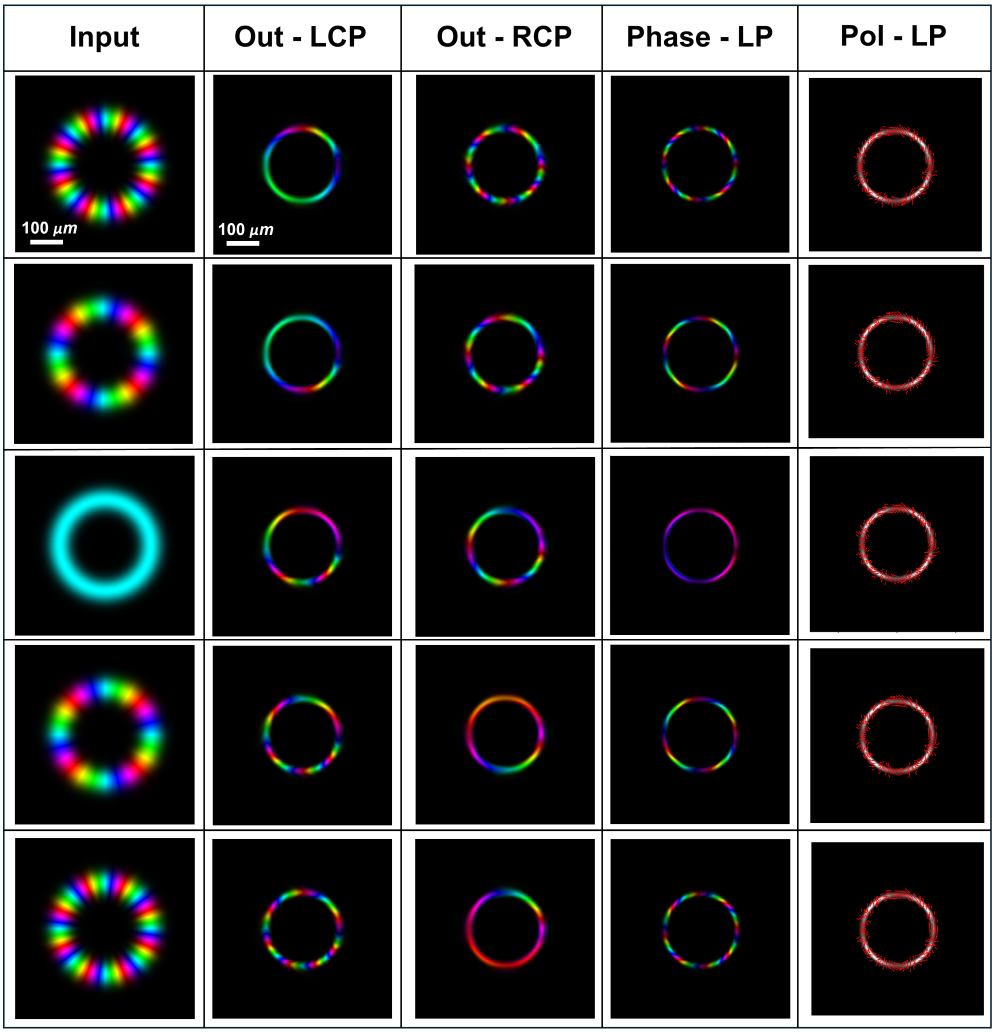

Fig. S7 First column depicts the impinging charged beam, modelled as a perfect vortex of waist $150\mu m$ and thick $50\mu m$ with a topological charge ranging from -8 to +8 at steps 4. The second and third columns represent the optical response left-handed and right-handed circularly polarized. It can be noticed that the phase of these two components is asymmetric (or unbalanced) when the metaoptics are illuminated with a charged beam (all rows except the third one). Colours refer to the phase range from 0 to 2π. The fourth and fifth columns represent the phase and the polarization pattern of the generated AV-PVB under the illumination of an horizontally polarized beam. It is easy to see that the vector beam's phase is the one of the impinging beam but the polarization pattern is the same.

S8 Non-separabilty of vector beams

The formal equivalence between non-separable vector beams and entangled quantum states enables the application of quantum concepts to classical light beams. Concurrence, a two-dimensional quantum entanglement measure, has been recognized as an effective metric for quantifying the non-separability of vector beams, serving as a vector quality factor [6]. The Concurrence (C) can be easily calculated through Stokes polarimetry as:

$$C = \sqrt{1 - \sum_{i=1}^{3} \frac{S_i^2}{S_0^2}} \tag{13}$$

where S_x are the four Stokes parameters [7]. We experimentally collect the six polarimetric projections of linear and circular components for each AV-PVBs generated from the illumination of a horizontally polarized light.

The average Concurrence calculated for all the 7 fabricated samples oscillates from 0.893 to 0.985. The high experimental values confirm the components' non-separability but slightly below 1. This can be motivated by little deviations from the HWP condition of metaatoms composing the metalens that lead to unwanted unconverted co-polarized light and non-perfect ideality of the optical components (linear polarizers and quarter-wave plates) used along the optical path.

Then, for completeness, we selected as a case study the metaoptics reported in Figure 4 a-b encoding and exponential phase gradient and extrapolated the Stokes parameters spanning the illuminating polarization (from circular to linear) by rotating the quarter-wave plate (QWP_1 in the optical setup of Figure 6) whose fast axis was initially fixed at $\pi/4$ radians to the horizontal axis before the metaoptics. The experimental values follow the theoretical prediction (Figure S8) confirming the ability to select the degree of non-separability by simply tuning the ellipticity of the impinging light.

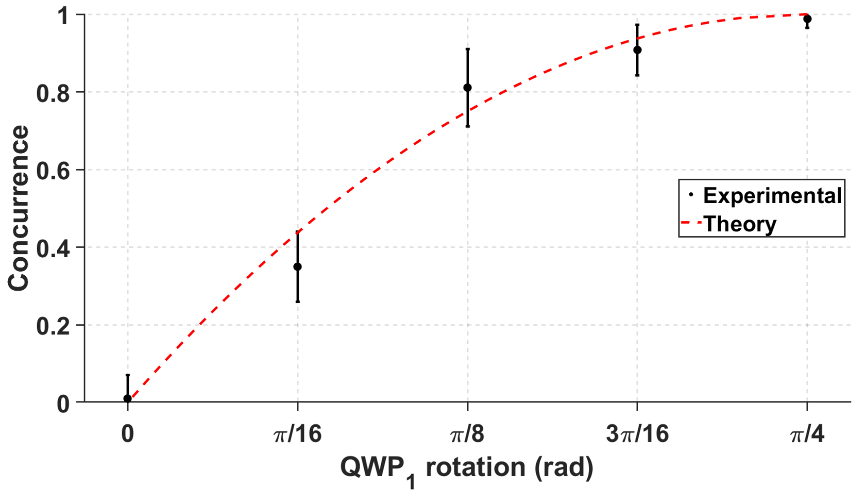

Fig. S8 Experimental (black) vs. theoretical (red dashed) Concurrence values varying the illuminating polarization from circular to linear, by rotating QWP_1.

S9 Experimental polarization state reconstruction

The Stokes parameters describe a polarization state by relating the amplitudes and relative phases of the x and y electric field components [8]. They can be conveniently expressed as the Cartesian coordinates of a point on the Poincaré sphere, representing the state of polarization (SoP), as follows:

$$
\begin{aligned}
S_0 &= \sqrt{S_1^2 + S_2^2 + S_3^2} \\
S_1 &= S_0 cos(2\chi) cos(2\psi) \\
S_2 &= S_0 cos(2\chi) sin(2\psi) \\
S_3 &= S_0 sin(2\chi)
\end{aligned}
\tag{14}
$$

where 2ψ is the azimuthal sphere coordinate and 2χ is the radial spherical coordinate. These parameters are also twice the angles that describe the orientation and ellipticity of the polarization ellipse. The relationship between the Poincaré sphere coordinates and the properties of the polarization ellipse is thus very simple, since the linear polarization states are located on the equator of the sphere ($\chi = 0$, $\psi \in [\pi/2, -\pi/2]$), and right and left circular polarization states positioned at the north and south poles, respectively ($\chi = \pm\pi/4$, $\psi = 0$). Elliptical polarization states are found on the remaining surface of the sphere ($\chi \in]\pi/4, -\pi/4[- \{0\}$. This connection to the polarization ellipse can be used to effectively describe the state of polarization (SoP) at each point (x, y) in the transverse plane of beam, allowing the complete experimental reconstruction of the SoP using the measured four parameters $S_0(x, y)$, $S_1(x, y)$, $S_2(x, y)$ and $S_3(x, y)$ by rearranging Eq.14 as:

$$
\begin{aligned}
\psi(x, y) &= \frac{1}{2} tan^{-1} \left(\frac{S_2(x, y)}{S_1(x, y)} \right) \\
\chi(x, y) &= \frac{1}{2} sin^{-1} \left(\frac{S_3(x, y)}{S_0(x, y)} \right)
\end{aligned}
\tag{15}
$$

In Figure S9, we reported the reconstructed state of polarization of each azimuthally-variant vector beam generated using the designed metasurfaces. Each of the generated beams has an extremely low level of ellipticity extremely low ($\chi \simeq 0$ along the whole rings, corresponding to light green colors) confirming the high level of Concurrence as reported in Section S8. On the other hand, the direction of the major axis of the polarization ellipses (corresponding to the direction of the linear polarization state as $\chi \simeq 0$) fits well the theoretical ones.

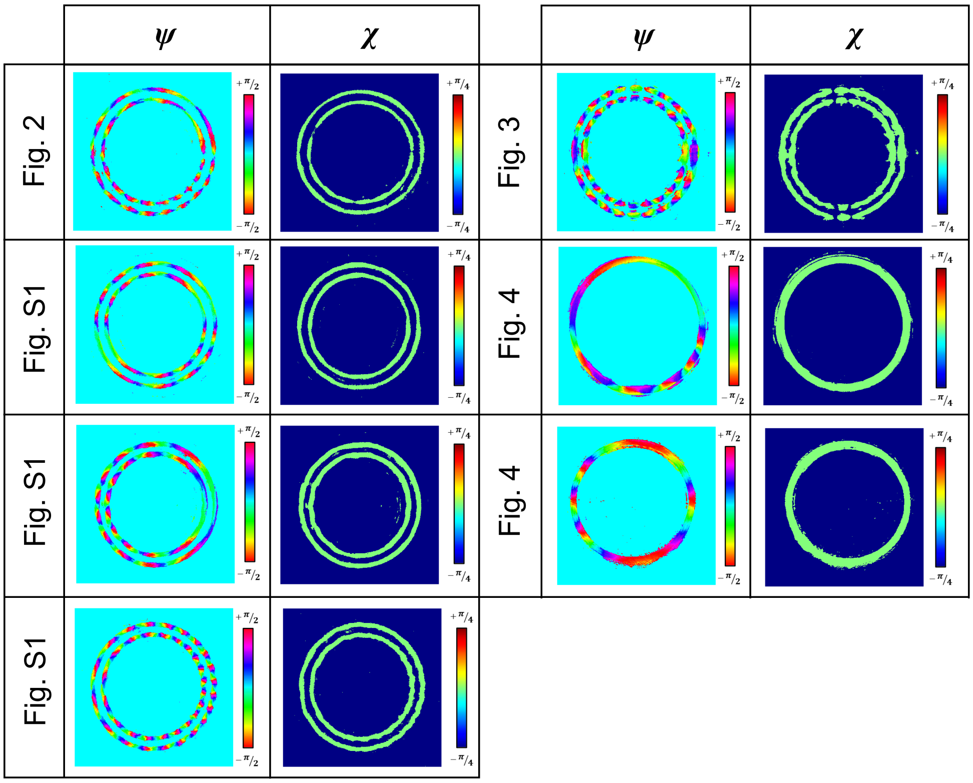

Fig. S9 The experimental polarization state retrieved from Stokes polarimetric projection is presented as maps of local orientation (ψ) and ellipticity (χ) of the polarization ellipse.

References

[1] Holleczek, A., Aiello, A., Gabriel, C., Marquardt, C. & Leuchs, G. Classical and quantum properties of cylindrically polarized states of light. *Optics express* **19**, 9714–9736 (2011).

[2] Vogliardi, A., Ruffato, G., Dal Zilio, S., Bonaldo, D. & Romanato, F. Dual-functional metalenses for the polarization-controlled generation of focalized vector beams in the telecom infrared. *Scientific Reports* **13**, 10327 (2023).

[3] Andrews, D. L. & Babiker, M. *The angular momentum of light* (Cambridge University Press, 2012).

[4] Vogliardi, A., Ruffato, G., Bonaldo, D., Dal Zilio, S. & Romanato, F. Silicon metaoptics for the compact generation of perfect vector beams in the telecom infrared. *Optics Letters* **48**, 4925–4928 (2023).

[5] Vogliardi, A., Bonaldo, D., Dal Zilio, S., Romanato, F. & Ruffato, G. Design, fabrication, and test of bi-functional metalenses for the spin-dependent oam shift of optical vortices. *Frontiers in Physics* **12**, 1381156 (2024).

[6] Żukowski, M., Laskowski, W. & Wieśniak, M. Normalized stokes operators for polarization correlations of entangled optical fields. *Physical Review A* **95**, 042113 (2017).

[7] Selyem, A., Rosales-Guzmán, C., Croke, S., Forbes, A. & Franke-Arnold, S. Basis-independent tomography and nonseparability witnesses of pure complex vectorial light fields by stokes projections. *Physical Review A* **100**, 063842 (2019).

[8] Born, M. & Wolf, E. *Principles of optics: electromagnetic theory of propagation, interference and diffraction of light* (Elsevier, 2013).
